# Cell Cycle-Driven Heterogeneity: On the Road to Demystifying the Transitions between “Poised” and “Restricted” Pluripotent Cell States

**DOI:** 10.1155/2015/219514

**Published:** 2015-04-05

**Authors:** Amar M. Singh

**Affiliations:** Department of Biochemistry and Molecular Biology, Paul D. Coverdell Center for Biomedical and Health Sciences, The University of Georgia, 500 D.W. Brooks Drive, Athens, GA 30602, USA

## Abstract

Cellular heterogeneity is now considered an inherent property of most stem cell types, including pluripotent stem cells, somatic stem cells, and cancer stem cells, and this heterogeneity can exist at the epigenetic, transcriptional, and posttranscriptional levels. Several studies have indicated that the stochastic activation of signaling networks may promote heterogeneity and further that this heterogeneity may be reduced by their inhibition. But why different cells in the same culture respond in a nonuniform manner to the identical exogenous signals has remained unclear. Recent studies now demonstrate that the cell cycle position directly influences lineage specification and specifically that pluripotent stem cells initiate their differentiation from the G1 phase. These studies suggest that cells in G1 are uniquely “poised” to undergo cell specification. G1 cells are therefore more prone to respond to differentiation cues, which may explain the heterogeneity of developmental factors, such as Gata6, and pluripotency factors, such as Nanog, in stem cell cultures. Overall, this raises the possibility that G1 serves as a “Differentiation Induction Point.” In this review, we will reexamine the literature describing heterogeneity of pluripotent stem cells, while highlighting the role of the cell cycle as a major determinant.

## 1. Introduction

Pluripotent stem cells (PSCs) have two defining characteristics, the ability to undergo indefinite self-renewal and the capacity to differentiate into the cells belonging to all 3 germ layers of the embryo: the mesoderm, endoderm, and ectoderm cell lineages [1]. Understanding the mechanisms that govern the processes of self-renewal and lineage specification continues to be a major focus for stem cell biologists, as these cells have tremendous potential for utility in cell-based therapies, disease modeling, and exploring the basic principles regulating early embryonic development and cell-fate commitment.

The classical paradigm describing the relationship between self-renewal and differentiation establishes that (1) a core set of pluripotency transcription factors are expressed to maintain self-renewal and suppress differentiation and (2) lineage-specific transcription factors become expressed to initiate differentiation following signaling cues [1]. Subsequently, upon differentiation, pluripotency factors are rapidly downregulated. This simple and elegant model, however, does not adequately explain the mechanisms describing the exit from pluripotency, and moreover, a number of recent studies challenge this classical view. First, several studies show that pluripotency factors may have a direct role in promoting differentiation to different cell lineages [2–4]. These studies raise the possibility that the so-called “pluripotency factors” have a role not only in maintaining self-renewal, but also in driving lineage specification to exit the pluripotent state. Secondly, recent studies in the field of reprogramming have demonstrated that you can reestablish the pluripotent state by the expression of lineage specifiers [5, 6]. In this model the expression of developmental factors suppresses alternate cell lineages promoting a pluripotent state. Thirdly, the recent identification of F-class pluripotent cells [7, 8], which have so far only been established during reprogramming, demonstrates that high and stably maintained expression of Oct4, Sox2, KLF4, and Myc promotes a self-renewing pluripotent cell. This F-class PSC is distinct from all other pluripotent cell types and expresses numerous lineage markers. Together these discoveries suggest that the traditional view and relationship between self-renewal and differentiation are not so clear-cut.

The classical notion of self-renewal and differentiation has also been challenged by the discovery of cellular heterogeneity within clonal stem cell cultures [9–11]. For example, several pluripotency factors have been shown to transition between “low” and “high” states in their expression levels during culture (see further details below). This heterogeneity of pluripotency factor expression during self-renewal indicates that the static expression of pluripotency factors is not a central requirement to maintaining pluripotency and inhibiting differentiation. Furthermore, the expression of developmental transcription factors has also been found to be transiently present during stem cell cultures. This so-called “metastability” of transcription factors during stem cell self-renewal is thought to be due to stochastic effects of signaling networks. While the importance of signaling networks is clear, recent studies by us, and others, now indicate that cell cycle positional effects also have a central role in promoting heterogeneity within stem cell cultures [12, 13].

## 2. Pluripotent Stem Cells and Their Atypical Cell Cycle

**Figure 1:**
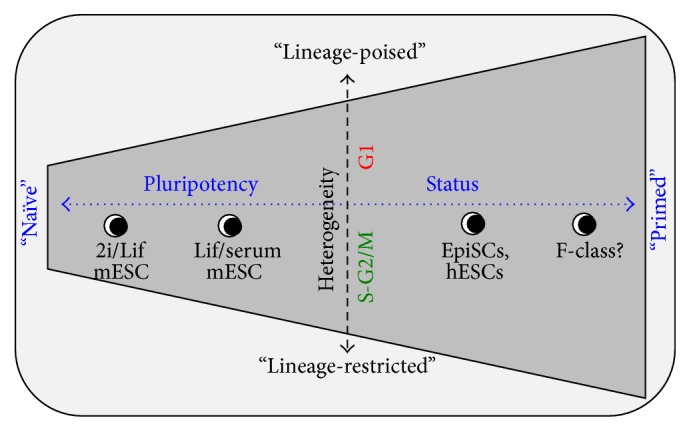
Hypothetical model depicting the relationship between heterogeneity, the cell cycle, and pluripotent cell types. Stem cells may transition horizontally on pluripotency spectrum (blue double-arrow) as they differentiate or dedifferentiate. As cells progress through the cell cycle, they transition on the heterogeneity spectrum (black double-arrow). The range of the heterogeneity, or metastable states, is shown in gray and increases as you move down the spectrum of pluripotency from naïve to primed cells. G1 cells exist in a “lineage-poised” state, while S-G2/M cells exist in a “lineage-restricted” state.

Numerous different types of pluripotent stem cells have been identified, either by direct isolation from embryos or by the reprogramming of somatic cells back to a pluripotent state [10]. The pluripotency status of these cells can range from the naïve/ground state pluripotent cells, such as mouse embryonic stem cells (mESCs) grown in 2i/Lif media [14], to the primed pluripotent stem cells derived from the epiblast, such as epiblast stem cells (EpiSCs) or human embryonic stem cells (hESCs, see Figure 1) [15–17]. By reprogramming, the F-class pluripotent state has also been identified [7, 8]. This state appears to be distinct from partially reprogrammed cells, expresses some but not all pluripotency markers, and generally expresses more lineage factors. Although it is unclear if this cell type exists* in vivo*, these cells are projected to be further down on the spectrum of pluripotency than the primed/epiblast-like cells. These data indicate that PSCs exist in a continuum of different cell states [18].

Regardless of their pluripotency status, PSCs typically have a unique cell cycle. We will only briefly consider this topic as numerous reviews examine this in considerable detail [19–21]. The cell cycle of mammalian PSCs is characterized by a short G1 phase and a large percentage of S phase cells [22]. Upon differentiation, the cell cycle undergoes a restructuring such that G1 lengthens and the number of cells in S phase is reduced [23, 24]. The molecular mechanism underlying this principle has been extensively examined with mESCs in traditional Lif/serum-containing media and to some extent in hESCs. Unlike somatic cells, which rely on mitogenic signaling from Fgf/Erk signaling, mESCs typically undergo differentiation from elevated Erk activity [25]. Instead mESCs rely on signals from PI3-kinase/Akt signaling, generated from factors such as insulin [14], to promote the cell cycle. Similarly hESCs also depend on PI3-kinase/Akt activity, which maintains Fgf/Erk signaling below a threshold required for differentiation [26, 27]. This notion is supported by studies using PTEN knockout mESCs, which proliferate rapidly [28]. Mouse PSCs do not appear to have an intact restriction (R) point as (1) E2F target genes appear to be stable throughout the cells cycle, (2) retinoblastoma protein (Rb) is stably hyperphosphorylated and inactive, and (3) Cdk2 expression and activity are in an elevated state [19]. On the other hand, hESCs do appear to have some Rb/E2F activity and may have a restriction point, but this is still not well understood [29–31]. Altogether these findings establish a mechanism for why pluripotent stem cells spend 50–80% of their time in S phase and only 10–20% of their time in G1. But how does this unusual cell cycle relate to cell specification?

**Figure 2:**
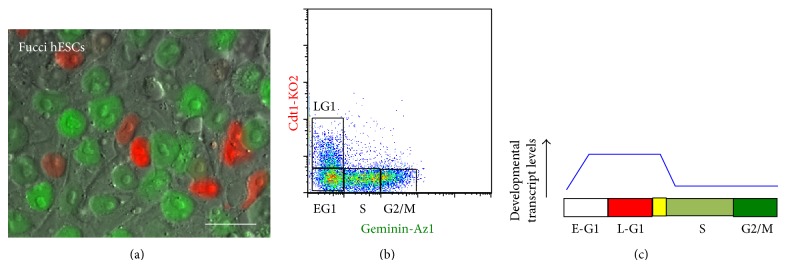
Fucci hESCs can be used to isolate cell cycle fractions from live cells. (a) Image of live Fucci hESCs. Bar: 25 *μ*m. (b) Flow cytometric analysis of Fucci hESCs, showing early-G1, EG1; late-G1, LG1; S phase, S; and G2/M-phases, G2/M. (c) Diagram showing that transcripts expressed from developmental genes have been found to be cell cycle regulated, peaking in G1 and downregulated in S phase.

Christine Mummery first described an intriguing relationship between the cell cycle and differentiation using embryonal carcinoma cells in 1987 [32]. Her work suggested that cells in G1 were more prone to initiate differentiation from signaling cues, such as retinoic acid, while cells in S phase were refractory to retinoic acid signaling. This discovery, that cells initiate differentiation from G1, has now been validated by several independent studies in human embryonic stem cells [12, 13, 33, 34]. Several recent studies utilize the Fucci (fluorescent ubiquitinated cell cycle indicator [35]) reporter system in ESCs to examine the relationship between the cell cycle, pluripotency, and differentiation [12, 13, 36–38] (Figure 2). Importantly, the Fucci system allows you to monitor and isolate cell cycle fractions from live cells, without the need for cell synchronization by chemical inhibitors such as nocodazole and aphidicolin. This provides a significant advantage over chemical blocks, as the cells remain unperturbed, thus minimizing artificial and nonspecific side effects. These studies indicate that G1 provides a “window of opportunity” where cells may commit to differentiation or continue to self-renew depending on signaling cues [12, 13, 39]. These studies provide a potential explanation as to why PSCs spend the majority of their time in S phase (i.e., to prevent unwarranted differentiation) and further suggest that G1 cells are in a “poised” state, such that they may commit to lineage specification. Together these studies establish the possibility that the cell cycle has a role in controlling cell state transitions and the “metastable” state of pluripotency.

Most, if not all, pluripotent stem cell subtypes appear to exist in a “metastable” state, where they undergo transitions between a “lineage-poised” state and a “lineage-restricted” state, which is dependent upon the cell cycle position (Figure 1). In this hypothetical model, I am making a clear distinction between the terminologies for “primed” versus “poised” pluripotent cells. A “primed” pluripotent cell refers strictly to those cells emanating from, or most reminiscent of, cells from the epiblast stage of the postimplantation blastocyst. Primed cells represent a window of time during embryonic development (or* in vitro* differentiation/reprogramming) on the spectrum of pluripotency. On the other end of the pluripotency spectrum are “naïve” cells, which are reminiscent of cells belonging to the inner cells mass of a peri-implantation blastocyst. “Poised” pluripotent cells refer to a cell that is ready to commit to differentiation and in the G1 phase. Poised cells express higher levels of lineage markers [12] and potentially reduced levels of pluripotency markers (discussed below). Poised cells are more responsive to differentiation-inducing signaling cues [12, 13, 32–34]. Furthermore, the epigenetic status and potentially chromatin structure within poised cells may be distinct or more amenable to rearrangement [12], permitting cell-fate specification. The converse of the poised state would be the “restricted” state, where cells exhibit reduced lineage marker expression and increased pluripotency marker expression and are primarily in S-G2 phases of the cell cycle [12]. These cells should be less susceptible to differentiation signaling cues and thus maintained in a protected state. Therefore, the terms “poised” and “restricted” refer to different positions on the spectrum of heterogeneity, which directly correlates with the cell cycle position. In this hypothetical model, both “naïve” and “primed” cells transition between “poised” and “restricted” states, as they progress through cell cycle stages (Figure 1). We will examine the evidence that support this model in the subsequent sections.

## 3. Heterogeneity of Pluripotency Factors

Numerous reviews have summarized the findings with regard to heterogeneity in pluripotent stem cell populations [9, 11, 40–44], so we will pay special attention to those studies that have uncovered a relationship with the cell cycle. Several pluripotency factors have been found to be heterogeneously expressed in PSCs, including Nanog [45–47], Rex1 [48], Oct4 [49], Stella [44], Esrrb [50, 51], KLF4 [52], and ZSCAN4 [53]. Three studies initially identified Nanog protein as being heterogeneously expressed during the traditional culture (Lif/serum) of mESCs [45–47]. Importantly, two of these studies showed by knock-in reporter systems that Nanog could transition between Nanog-low and Nanog-high states [46, 47]. These findings were confirmed by others and used in computational modeling to predict the mechanics of these gene fluctuations [54, 55].

Several mechanisms have been attributed to the transition of Nanog between low and high states. One of the more intriguing models suggests that* Nanog* transcription is regulated at the allelic level [56]. Using fluorescent reporters under control of the Nanog promoter, Miyanari and Torres-Padilla found that* Nanog* is monoallelically expressed in mESCs in Lif/serum media but then switches to a biallelic expression as cells transition back towards a naïve stem cell state in 2i/Lif media. While initial single-cell RNA-sequencing studies failed to confirm these findings [57], other single-cell assays have also found fluctuations in pluripotency markers, including* Nanog* [18, 51]. Knock-in fluorescent proteins fused to Nanog also are not supportive of monoallelic expression variations [58]. However, half-lives of monoallelic transcript and protein, along with protein and RNA stability, may be considerably different [41, 59], although* Nanog* RNA and protein levels appear to correlate well [60]. Allelic expression patterns may not be the sole determinant of* Nanog* heterogeneity, however, as heterogeneity of* Nanog* may persist in 2i/Lif media [61] where biallelic expression occurs. Other possible mechanisms, such as the cell cycle and signaling environment, may then have a role in controlling* Nanog* heterogeneity.

What is the relationship between Nanog and the cell cycle? Our initial study, which identified Nanog heterogeneity and suggested that* Nanog*-low and* Nanog*-high mouse ESCs could interconvert in Lif/serum media [47], evaluated the gene expression patterns between* Nanog*-low and* Nanog*-high cells using microarrays. Interestingly, we found that numerous cell cycle genes were elevated in the* Nanog*-high cells. These included* Cyclin B1*,* Aurora Kinase B*,* E2F1,* and* Wee1*, which are genes that are well known to be elevated in S and G2 phases of the cell cycle.* Nanog*-low cells, on the other hand, expressed* CDK inhibitors 1C* and* 2B* [47], which are commonly found in G1. When* Nanog*-low cells were replated at limiting dilution concentrations, nearly all cells were able to reestablish colonies with cells expressing the* Nanog* reporter [47]. While this reduces the likelihood that contaminating differentiated cells exist within the* Nanog*-low subpopulations, this caveat however cannot be completely excluded. Overall, these observations raise the hypothesis that* Nanog* expression may fluctuate during the cell cycle in mouse ESCs grown in Lif/serum. We found that* Nanog*-low cells ranged from 5 to 10% of the total culture, which would be consistent with the percentage of cells in G1 [47]. In agreement with these findings, work by Macarthur and colleagues [55] demonstrates that the depletion of* Nanog*, using an inducible system, leads to an accumulation of cell cycle checkpoint genes, such as* CDK inhibitors 1A*,* 1B*, and* 2A*, suggesting an arrest in G1 [55]. Importantly, the reintroduction of* Nanog* led to a loss of the cell cycle checkpoint genes. Recently,* Nanog* expression, but not* Oct4*, has been shown to be dynamic during the cell cycle in mESCs [62]. Although the data relied on chemical blocks, which may have unwarranted effects, further confirmation is necessary.

In hESCs, Nanog has been shown to directly control the expression of cell cycle genes,* CDK6* and* CDC25A*, and therefore regulate the G1 to S phase transition [63]. In addition, the pluripotency network consisting of Oct4, NANOG, and SOX2 was also found to regulate the* miR-302* cluster [64]. Loss of* miR-302* led to an increase in G1 cells, which was mediated by an accumulation of Cyclin D1. Together these findings suggest that the pluripotency network controls the cell cycle transitions and the length of G1. By using the Fucci system in hESCs, we have not observed any changes to* NANOG* transcript or protein levels during the cell cycle [12]. However, given the proposed differences in* Nanog* allelic expression between mESCs in traditional Lif/serum media and epiblast-like cells [56], and the differences in signaling requirements for these pluripotent cell types, this raises the possibility that* Nanog* may be cell cycle regulated in peri-implantation blastocyst embryos and stabilized at the postimplantation epiblast stage. In this scenario, the cell cycle position may also influence the allelic expression patterns. Another possibility is that stochastic promoter activity associated with* Nanog* in mESCs [60] becomes stabilized as cells move further down the continuum of pluripotency, which may be important for Nanog's role in lineage specification [2, 3]. In either case, more studies examining the relationship between Nanog and the cell cycle are warranted.

Other pluripotency factors, which are expressed heterogeneously, have also been suggested to control the cell cycle. For example, the deletion of* Rex1* in mESCs was found to result in increased expression levels for* Cyclin D2* and* Cdk inhibitor 2B* and decreased* Cyclin E2* [65], consistent with increased number of G1 cells and a decreased number in S-G2. Interestingly,* Nanog*-low cells exhibit reduced expression levels for* Rex1* [47], and* Rex1*-low cells were found to have reduced expression levels for* Nanog* [48]. Altogether these studies may suggest the occurrence of a G1-subpopulation that is low in pluripotency marker expression. Finally, another potential pluripotent factor that may regulate the cell cycle is KLF4. In particular KLF4 is thought to regulate the cell cycle checkpoint controls including G2/M in nonpluripotent cell types [66]. However, no studies have yet examined the role of KLF4 in controlling the cell cycle in pluripotent populations.

Other studies have also identified a relationship between the pluripotency marker SSEA3 and the cell cycle in hESCs [67]. Bhatia and colleagues used SSEA3 to isolate SSEA3− and SSEA3+ subpopulations and subsequently performed cell cycle analyses. Importantly, SSEA3− populations were heavily enriched in G1 cells, while SSEA3+ populations were enriched in S-G2/M cells, and these populations could interconvert between each other. Furthermore, both Nanog and Oct4 protein (but not transcript) were elevated in the SSEA3+ population, over the SSEA3− population. Overall, these studies support the hypothesis that pluripotency markers are cell cycle regulated and become diminished in G1 to establish a “poised” pluripotent state. This provides a plausible explanation for their apparent heterogeneity of pluripotency factors under certain signaling conditions.

## 4. Heterogeneity of Developmental Factors

Background expression levels of developmental factors can be readily identified during the culture of PSCs. This background level, however, is not usually ubiquitous in all cells and instead heterogeneously expressed in only a small subset of cells. The heterogeneity of developmental factors has been observed for* Gata6* [12, 47],* Sox17* [12, 68],* FOXA2* [12],* HEX* [69],* Hes1* [70], and* BRACHYURY* [26]. Furthermore, single-cell expression analysis in hESCs has identified lineage-primed subpopulations, in which developmental genes are expressed [71].

While signaling network dynamics clearly have a role in promoting heterogeneity among developmental factors (discussed below), we have found that the cell cycle position has a direct role [12]. By using the Fucci system in hESCs and performing RNA-sequencing in cell cycle fractions, we observed that numerous developmental genes for all 3 germ layers were cell cycle regulated, and this cell cycle regulation persisted during differentiation (Figure 2). Moreover, most of these developmental genes peaked in the G1 phase of the cell cycle. This is consistent with cells initiating their differentiation from the G1 phase [12, 13, 32–34] and establishes G1 as the poised pluripotent state.

One important question that remains is as follows: do these developmental genes have a role in regulating the cell cycle or reorganizing the cell cycle structure as cells differentiate? While this is difficult to address, since deletion of these genes can sometimes block differentiation, this will be pertinent to further understand the relationship between lineage specification and the cell cycle. Further global studies that identify the target genes for early developmental regulators during PSC differentiation should shed light on this question.

## 5. Signaling Networks and Their Effect on Heterogeneity

Stochastic activity of signaling networks is considered to be the major contributing determinant to cellular heterogeneity in stem cell cultures. Heterogeneity of pluripotency and developmental factors in mESCs were initially observed in serum-based media, where signaling effectors are indeterminable. However, when mESCs were grown in defined media conditions in the presence of Mek/Erk and Gsk3 inhibitors (2i/Lif), a considerable reduction in heterogeneity has been observed [14, 72]. In particular, the background expression of developmental genes for all germ layers is reduced. Furthermore, there is a significant loss of H3K27me3 and fewer bivalent domains altogether. Based on these findings, mESCs in 2i/Lif media are considered to be in a naïve or ground state (Figure 1). Given the importance of Erk activity in development [25] and Wnt signaling in mESC self-renewal [73–76], the reduction of developmental gene expression in 2i/Lif media is not too surprising. However, all heterogeneity is not lost in 2i/Lif media, as* Nanog* still fluctuates [61]; some developmental genes such as GSC are still expressed [72], and developmental reporters for Hex are still heterogeneously present [11, 69]. Together these data would suggest that other factors besides cell signaling, such as a cell's position within the cell cycle, are also of critical importance.

Unlike Erk signaling, the role of Wnt signaling in ESC self-renewal and differentiation is more complex, with some findings suggesting it promotes self-renewal and other findings suggesting it promotes differentiation. This may be directly due to concentration dependent effects of Gsk3 inhibitors used to mimic Wnt signaling. At high concentrations of Gsk3, *β*-catenin-dependent signaling is heavily activated which promotes differentiation [26, 77, 78], while at lower doses Myc or *β*-catenin/Tcf3 complexes are stabilized, which promotes self-renewal. Exogenous expression of Wnt3a promotes the expression of pluripotency and endoderm genes, resulting in increased heterogeneity mediated through Tbx3 [79]. Furthermore, *β*-catenin has also been found to fluctuate in mESCs and contribute to Nanog heterogeneity [80]. These data clearly indicate an important role for Wnt signaling in promoting heterogeneity of stem cells.

In hESCs, endogenous WNT signaling is well documented to promote heterogeneity within stem cell cultures [26, 81, 82], and inhibition of WNT signaling with antagonists, such as DKK1 and XAV939, significantly reduces the heterogeneous expression of developmental genes such as* GATA6*,* SOX17*, and* BRACHYURY*. Interestingly, while we have found that* GATA6* and* SOX17* are cell cycle regulated,* BRACHYURY* is not, which may indicate that there are multiple layers of heterogeneity [12]. One explanation for this may be due to the requirement for specific *β*-catenin cofactors necessary for transcriptional activation. For example, in some cases SMAD2,3 may be a corequirement for *β*-CATENIN, such as for endoderm genes like* MIXL1* [26], while other genes may require a different set of cofactors. Recent work has shown that SMAD2,3 shuttles in and out of the nucleus in a cell cycle-dependent manner, and this is dependent upon CDK4/6 activity [13]. This provides one explanation for the cell cycle regulation of* GATA6* and* SOX17* transcripts, and cell cycle-independence of* BRACHYURY* transcript, as* GATA6* and* SOX17*, but not* BRACHYURY*, may be dependent upon ACTIVIN/SMAD2,3 signaling.

A recent study by Hough et al. utilized single-cell sequencing from subpopulations of cells separated based on cell surface molecules, GCTM2, CD9, and EPCAM, to examine heterogeneity in hESCs grown under different self-renewal media conditions [71]. In addition to observing heterogeneity with pluripotent and developmental factors, they also observed heterogeneity among signaling molecules, and especially those belonging to the TGF-beta superfamily. Importantly, they confirmed this heterogeneity at the protein level by immunostaining. This provides evidence that the activity of signaling networks may vary on a cell-to-cell basis. Since the cell cycle has a role in controlling heterogeneity, one hypothesis here is that the cell cycle may control signaling pathway activities, but further studies are needed here. It should also not be overlooked that within the TGF-beta superfamily, competing Nodal and BMP signals can regulate the heterogeneity of pluripotency factors, such as* Nanog* [83].

We have found that the position of a cell, within the cell cycle, directly effects heterogeneity caused by WNT signaling and ERK signaling [12]. The inhibition of WNT activity, by DKK1, or MEK/ERK activity, by U0126, significantly reduces heterogeneity of* GATA6*,* SOX17,* and* FOXA2* found in G1 cells. Again, these data raise the hypothesis that a cell becomes poised in G1, which makes them more permissive to respond to differentiation-inducing signals by WNT or ERK. The end result is the heterogeneous expression of developmental factors. Overall, it is clear that signaling networks are directly required for promoting heterogeneity within stem cell subpopulations, where in many cases the subpopulation is determined by the cell cycle position.

## 6. Conclusion and Future Perspectives

It is now becoming increasingly clear that heterogeneity is a natural part of pluripotency [12, 84]. In many cases, this heterogeneity is a direct result of a cell transitioning through different phases of the cell cycle. After a cell completes mitosis and enters into G1, it becomes “poised.” This cell-poising likely reflects different epigenetic and chromatin configurations, which allows the cell to be permissive to either continue self-renewing or commit to lineage specification. This cell-fate choice is entirely dependent on the signaling environment. As the cell further progresses into S phase, the cell becomes less responsive to differentiation cues and is now in a “restricted” state. Why a cell must be in a “restricted” state in S and G2 is less clear, but this may have to do with temporal control and the importance of maintaining error-free gene duplication. G1 is often the focus of a cell's decision making center as the restriction point [85] and also the replication timing and the chromatin architecture [86, 87] are established here. Therefore we may consider G1 to be the “Differentiation Induction Point” for pluripotent stem cells.

Examining the molecular determinants controlling heterogeneity of pluripotent stem cells has been considerably helped by several studies utilizing single-cell RNA-sequencing [51, 57, 71]. However, the hurdle of technical variability with this methodology should not be overlooked [88]. Also, the cell cycle position can also lead to unwanted noise and can be reduced through computational algorithms [89]. Nonetheless, the utility of single-cell approaches to understand that heterogeneity is clear. Obtaining a full appreciation of the complexities with regard to the dynamic nature of epigenetic marks, at the single-cell level, remains a necessary hurdle yet to be overcome. Indeed we have found that 5-*hydroxymethylcytosine* (5hmC) is dynamic during the cell cycle [12]. Recent advances with single-cell DNA methylation approaches [90, 91] and single-cell chromatin structure techniques [92] will be essential for further studies in heterogeneity, but methods to determine genome-wide distribution for histone modifications in single-cells still need to be developed.

The role of the cell cycle in controlling posttranscriptional regulation of genes has also not been elucidated. We have identified two proteins, FOXA2 and SOX17, which oscillate during the cells cycle [12]. How these proteins are turned over in a cell cycle-dependent manner remains to be determined. Proteomics-based approaches aiming at the global identification of proteins that fluctuate during the cell cycle from pluripotent stem cells would present additional insight. Furthermore, the identification of posttranslational modifications, and their targets, that occur in a cell cycle-dependent manner will be important for future studies. Altogether, “omics” approaches will be critical in the future to understand the underlying mechanisms regulating heterogeneity and cell-fate specification.

In this paper, I have outlined the current understanding of the relationship between heterogeneity, the cell cycle, and lineage determination. It is clear that both the cell cycle position and the signaling networks converge to promote heterogeneity of developmental factors. While there is some evidence that pluripotency factors may oscillate during the cell cycle, further studies are still needed here. Clearly, however, pluripotency factors have a role in regulating cell cycle progression. In summary, the cell cycle position provides a direct explanation for metastability in stem cell cultures, which is necessary to regulate cell-fate commitment.
